# Reflex Ocular and Facial Symptoms of Nasal Disease

**Published:** 1890-06

**Authors:** Edward S. Peck

**Affiliations:** Visiting Surgeon to Charity Hospital; Consulting Surgeon to Bellevue Hospital, Out-door Department; Ophthalmic Surgeon to the Montefiore Home for Chronic Invalids; Member of the New York Pathological Society; Fellow of the New York Academy of Medicine, etc.


### International Dental Journal.

Vol. XI. June, 1890. No. 6.

### Original Communications.[1]

### REFLEX OCULAR AND FACIAL SYMPTOMS OF NASAL DISEASE.[2]

BY EDWARD S. PECK, A.M., M.D.,

Visiting Surgeon to Charity Hospital; Consulting Surgeon to Bellevue Hospital, Out-Door Poor Department; Ophthalmic Surgeon to the Montefiore Home for Chronic Invalids; Member of the New York Pathological Society; Fellow of the New York Academy of Medicine, etc.

The subject of reflex neuroses or of distant reflexes of the nervous system, has always been an interesting one; and it will so continue, as long as there remains any region of disease which fails of coming under the law of localized areas of nervous force, or of being subordinated to distinct nervous impulse. To be able to discuss intelligently the *fons et origo* of symptoms of disturbances of one organ, by means of disease located in another organ, possibly quite remote, is quite in the scope of the topic under consideration. It rather disarms our faith in the old physiological law of direct nerve-irritation to be told of pathological phenomena appearing in a part remote from the source of irritation; but you will agree

[1] The editor and publishers are not responsible for the views of authors of papers published in this department, nor for any claim to novelty, or otherwise, that may be made by them. No papers will be received for this department that have appeared in any other journal published in this country.

[2] Read at the stated meeting of the New York Odontological Society, February 18, 1890.

with me in the truth of this possibility by multiple illustrations taken from symptoms in the face and mouth of disease that is purely, and without question, dental. On this subject the literature is quite meagre. I am astonished to find that twenty years ago a most able authority[1] in dental literature hinted at dental reflexes, when he said that neuralgia is not a disease, but simply a phenomenon, or the expression of an immediate or distant lesion.

The first inquiry in neuralgia, he writes, is to search for the cause, which is by no means evident; it exists, and in many instances nothing short of a thorough search will reveal it; when, to argue effect from cause, we will find that principles must be deserted, physiological theories must be abandoned, even anatomical relations and connections must be forgotten; and the neuralgic expression must be treated "with the hope of an accidental success."

As graphic examples of the phenomena of distant and peripheral neuralgias, there may be cited otalgia, hemicrania, or even sciatica, as a result of an exposed tooth-pulp, or of pressure from an exostosis. It is undoubtedly a familiar fact that the extraction of a canine, or bicuspid, or molar tooth, relieves, as with this author, neuralgia of the face, odontalgia occurring in consequence of menstrual irregularities, megrim, and even neuralgia of the nates and thighs. As further instances of dental reflexes, and more pertinent to the scope of this paper, may be mentioned the neuralgias found in the infra- and supraorbital nerves, the globe of the eye, the temples, and particularly in a spot near the vertex, a little to that side of the median line on which the affected tooth is found. Catarrhal affection of the middle ear has been caused by carious teeth; so, too, amaurosis by the crowding of teeth. All these and more are cited as examples of dental reflexes.

There are, however, as every dental surgeon knows, many instances of distant neuralgias, which neither extraction nor filling of the properly-selected tooth will alleviate.[2] These instances can only be explained, according to Von Recklinghausen, by the irritation of the spheno-palatine ganglion and superior maxillary nerve. The alveolar border of the teeth possesses a rich kingdom of nerves,

---

[1] Garretson, "Diseases and Surgery of the Mouth, Jaws, etc." Philadelphia: Lippincott & Co., 1869.

[2] Ziem, "Bedeutung d. Zahnkrankheiten für d. Entstehung v. Nasenleiden." *Allg. Med. Centr. Zeitung*, September 2, 1885, p. 1117.

—the anastomosing filaments of the anterior and posterior dental nerves. How often do we see teething children, and children suffering from caries of the upper back teeth, who have at the same time discharges from the nasal mucous membrane, with swelling of the inferior turbinated bones and adjacent parts of the septum nasi, and with possible elevation of temperature?

In isolated cases the same connection is found between dental cysts, alveolar swellings, pathological growths from the teeth into the buccal cavity or into Highmore's antrum, abscess-formations of tooth-roots, etc., with swellings of the nasal mucous membrane, of the lips, or cheeks. In some of these isolated cases the only way to destroy the persistent neuralgias, or to prevent their insidious recurrence, is by means of the galvano-cautery, or chemical caustic to the nasal mucous membrane.

So far as I know, the first one to call the marked attention of specialists to the dependence of conjunctival catarrh upon nasal and pharyngeal catarrh was Bresgen,[1] in 1881. Arlt, of Vienna, had dwelt upon the close connection between stricture and chronic inflammation of the lachrymal duct and diseases of the mucous membrane of the nose for many years in his admirable clinical hours with his students. From earliest time it has been known that, if an eye were injured, or suffered from the lodgment of a foreign body upon any of its coats, more especially upon the cornea, the first phenomenon is a one-sided "snuffle," or a sudden rhinorrhæa. This is not merely an increase of tears, because a sudden hyperæmia of the nasal mucous membrane of the same side follows. The introduction of a Eustachian catheter, in its passage over the floor of the nostril, and opposite the anterior and middle parts of the inferior turbinated bone, is immediately followed by a flow of tears from the corresponding eye; after the catheter has passed this short tract, the sensation produced by it is confined to the posterior part of the nasal strait, and is not accompanied by any ocular symptoms. This gush of tears cannot be explained by a stoppage of the nasal end of the lachrymal duct, for the tears are not conveyed with such rapidity into the duct; and, moreover, the overflow on the side of the catheter-introduction is immediately succeeded by a flow of tears from the other eye.

These and similar physiological phenomena led Bresgen, Hack, Ziem, and later, Gruening, Rumbold, Rothholz, Bosworth, etc., to observe certain ocular symptoms consequent upon nasal disease.

---

[1] Der Chronische Nasen u. Rachen Katarrh, 1881, Band I.

Hack's paper attracted universal attention, and, while it was at first received with quiet favor on account of its source, soon invited unsparing criticism and strong opposition from the Berlin Medical Society, where it was first presented. Hack found a large number of ocular disturbances that would not yield to proper local treatment; and, in looking about for a definite cause, found not infrequently a series of nasal symptoms, which one author[1] has grouped together under the homely phrase of "irritable nose." These symptoms were stuffiness of one or both nostrils, usually of the one corresponding to the affected eye; a catarrhal discharge; local nasal irritation, usually described as in the lower and middle parts of the nasal fossa, and inability to breathe freely through the nostril. Exploration with the nasal probe produced an engorgement of the mucous membrane of the anterior extremity of the inferior turbinated bone, and sometimes a puffing of the corresponding portion of the septum. This touching with the probe was like touching a puff-ball. It was found that the submucous cavernous tissue had undergone irritation, had become spongy, enlarged, and erectile.

The ocular disturbances physiologically produced by this manœuvre were lachrymation, fear of light, known by the term photophobia, itching of the lids, a more or less bloodshot appearance of the conjunctiva of both lid and ball, and sometimes an injection around the cornea; more or less pain accompanied these symptoms. Hack regarded these disturbances as paroxysmal and reflex. The question of reflex irritation, therefore, was not so much one of enlargement and engorgement of nasal tissue and of obstruction of the nostril, as of the remote effect upon the eye. This is also illustrated by the familiar effect of sneezing, of the sudden entrance of foreign bodies into the nose, of twitching out hairs from the nostril, etc., whose prompt effect is to suffuse the eyes with tears, and to produce ocular congestion.

It would be improper to consider the pathological disturbances of the eye, as the possible result of nasal disease, without excluding certain groups of diseases which must be regarded as determining their own manifestations; as, for instance, all forms of direct traumatism; all scrofulous and syphilitic diseases, whose import is shown quite as much in the skin, glands, teeth, etc., as in the nose or eye, and which may be expressed in one or all of these different systems simultaneously, or consecutively, without any reflex element whatever. For instance, the occurrence of a one-sided ophthalmia,

---

[1] Gradle, *Archives of Ophthalmology*, December, 1887, p. 391.

of necrotic ulcers of the cornea, or of an iritis, during an attack of scrofulous rhinitis, or of ozæna, or of nasal ulcers and eczema, cannot be regarded as reflex; it is also a well-known fact[1] that wounds of the cornea of the eye are very apt to become suppurative, necrotic, and perforating during the contemporary existence of an ozæna, but it is not reflex. A more intelligent view of such coincidences is that they are mutually dependent upon a central systematic cause, and their removal depends upon a persistent fight with this central lesion. It is doubtful, in such coincidences, which disease is the starting-point of the other; in fact, it is best to regard them as independent of each other, and to treat them simultaneously as symptoms of a general cause. Every physician has cases in which such a connection or coincidence exists; and in the elimination of those that have no connection with each other lie the genius of diagnosis and the success of treatment.

The pathological disturbances of the eye and face which may be regarded as belonging to the category of reflex symptoms, due to nasal disease, are as follow: lachrymation; congestion of the eyeball and lids; floating bodies in the vitreous humor of the eye; phosphorescences in the eye; itching and scratching of the inner surface of the lids; eczema at the margin of the lids; a fulness of the lids just behind the bases of the cilia, and to be contradistinguished from the puffy œdema of albuminuria; eye-strain, or eye-tension, described by patients as if a tight band, all too short, held both eyes together, or as if a band fixed the eye in the orbit, making its motions painful; weakness of sight, known by the term asthenopia, the result either of a weak muscular apparatus, or of imperfect accommodation for near objects; headache, either temporal, vertical, frontal, or cervical, chiefly the two last varieties; spasm of the lids; choreic movements of the lids and eyeballs; neuralgia of the supraorbital, infraorbital, and canine regions of the face; pain, tenderness on pressure, and swelling over the region of Highmore's fossa; functional epilepsy, manifestly due to reflex influence;[2] nausea and gastric disturbances; a diseased condition of the upper digestive tract; uterine disorders; genito-urinary affections; perversions of smell, taste, and hearing, as well as of sight; laryngeal cough; bronchial asthma; megrim; melancholy, etc. Since Voltolini first observed the reflex connection between nasal polypi and asthma,

---

[1] Rothholz, *Deutsche Medicinische Wochenschrift*, December 29, 1887, p. 1123: "Ueber die Beziehungen von Augenkrankh. zur Nasen-affectionen."

[2] Elsberg, *Archives of Laryngology*, 1883, iv. p. 253.

between sneezing and asthma, between sneeezing and spasmodic cough, a great deal of attention has been paid to laryngeal and nasal reflexes. In 1880, Zuckerkandl, of Vienna, made a careful examination of one hundred and fifty skulls, with reference to the existence of catarrhal inflammation in the cranial sinuses. He found that the sinus, or antrum, of Highmore, is most frequently affected with catarrhal processes; after that the sphenoidal sinuses; and *least frequently of all*, the frontal sinuses.

The following cases will serve as illustrations of some of the reflexes named. They are selected from a varied array, for the emphasis of special symptoms.

A married lady of thirty years, mother of one child by a normal delivery, succeeding to a miscarriage by two years, occupation that of care of an easily-managed household, with time and inclination to cultivate herself, to a limited extent, in music and literature, enjoying otherwise good health, came to me in October, 1888, complaining of blurred vision, and floating specks chiefly in the left eye; itching and scratching of left lower lid; pain between the eyes; a cincture-band around the forehead; headache after using the eyes for sewing and reading, and on the following nights an aggravating insomnia. Correction of a hyperopic astigmatism with proper glasses brought vision to the normal, and an eye-drop of one-half per cent. of cocaine-hydrochlorate and one grain of zinc-sulphate improved the congested and granular condition of the lids. The remedies were sufficient for a time. Soon, however, the frontal ache and cincture-band, with the insomnia, asserted themselves more strongly, and my attention being directed to her nose and throat, I found a catarrhal rhinitis, chiefly of the right nostril; a deviation of the septum, partly of osseous and partly of cartilaginous tissue, into the same nostril; a puffing of the mucous membrane of the right wing of the nostril, opposite the septal malformation: the two entirely filled up the middle and superior meatuses of the nostril, but the nasal probe could be easily passed between them to the post-pharyngeal wall; the puffy alar hypertrophy was very sensitive to the touch, and fairly rough usage of the probe immediately produced a flow of tears, with a pericorneal injection of the same eye. Under the belief that cocaine ought to assist in establishing a connection between the nasal disturbances and the ocular symptoms, a four-per-cent. solution of cocaine was freely used, with the effect of a partial retraction of the alar hypertrophy, but with only slight abatement of the ocular symptoms. A systematic treatment of cocaine-spraying and pencilling was then used, together with Dobell's solution of borax, bicarbonate of soda, carbolic acid, and glycerin, with considerable relief of the nasal sensitiveness and of the ocular symptoms. It was not, however, until the osseo-cartilaginous spur was freely removed with a saw, that freedom from distressing symptoms could be asserted. This was done with a ten-per-cent. solution of cocaine, applied for fifteen minutes with a camel's-hair pencil, and on a cotton probe. The sawing was with an upward movement, and brought away quite a large triangular spur of the septum. Frontal and ocular symptoms disappeared, followed by complete absence of insomnia.

This case may be regarded as a fair example of distinct nasal and ocular diseases, at once dependent upon, and independent of, each other. The refractive error of hyperopia was certainly distinct from the nasal disease; and, following the law of such optical anomalies, it was due to an anatomical defect of the eyes. But its careful correction did not relieve the patient of the reflex ocular symptoms, whose origin was in the nose, and which finally disappeared with a removal of the offending nasal obstructions. In the simpler forms of nasal mucous-membrane hypertrophy a resort to operative procedures is usually unnecessary. Cocaine, in a two- or four-per-cent. solution, when well borne, with Dobell's spray, or Seiler's antiseptic pastilles, and, if necessary, chromic acid as a caustic application, will in a large number of cases suffice to effect a cure. As to operative procedures, it is to be remembered that the nostrils are not symmetrical in two-thirds of all the cases, and that not every deviated septum is to be "shaved off," or "sawn off." The practice of "rifle-boring" a nostril, or making it as "clean as a gun-barrel," is to be condemned. It is to be borne in mind that the nose receives the first air that is destined to pass into the lower respiratory organs; and sufficient area is needed in either nostril to allow of its free passage, otherwise the mouth must be opened to assist in respiration, and thereby the faulty habit of mouth-breathing will be acquired; it is also to be borne in mind that the nose is the organ of smell, and destruction of excessive nasal tissue, especially of that of the bony septum, is not in the line of good surgery, on account of its interference with this function.

Another case is as follows: A man of forty years, of large physique and bony frame, never having had syphilis, and apparently in perfect health, by occupation a teller in a bank in the northern part of this State, had suffered for some years from conjunctival catarrh of both eyes, and had worn glasses with the idea of relieving eye-strain while at work upon the books. He was a mouth-breather, with enlarged tonsils, chronic follicular pharyngitis, false noises heard in both ears, slightly but not noticeably deaf, had central frontal headache, especially at the close of the day's work; in addition, he had hay fever for a limited time during the summer. Cocaine in weak solution had been used upon his eyes, giving temporary relief. Having fallen into good hands, he found himself the unfortunate possessor of "sensitive areas" in each nostril, which had been touched with the red-hot platinum wire. I found that, though there was an improvement in the size of the nasal straits by the removal of the alar hypertrophies, there remained a large deviated septum into the left nostril, which, at its middle and posterior parts, was sensitive to the touch of the probe, and somewhat puffy. Under thorough local anæsthesia this was sawn from below upward with some difficulty, and the major part of the obstruction was removed. The result has been a return to normal breathing by the nose, an abeyance of the false aural sounds and of the stuffy feeling of the ear, eye-tension and discomfort have disappeared, and glasses have been laid aside.

I shall cite one more case of perhaps greater dental interest, wherein relief failed to come by means of the methods heretofore indicated, and then close these observations upon this interesting topic.

A New York merchant of nearly thirty years of age, whose father is engaged in large manufacturing interests, and who himself was engaged in buying raw materials, in office-work, and in shaping national legislation in regard to import duties, whose duties, therefore, were varied and responsible, but not specially trying to his eyes, was sent to me in January, 1887, by a dentist, with a history, concisely stated, as follows: Patient had had intermittent pain in the fifth pair of nerves, chiefly over the superior dental nerve, and in the canine fossa, running up through the superior lateral incisor teeth; this pain was now on one side and now on the other; bilateral temporal pain and discomfort, chiefly over the right temple, on which side was chiefly located the canine pain; pain behind both eyeballs. In March, 1886, he fell twenty feet in the gymnasium of one of the athletic clubs of the city, and broke off both lateral incisors of the upper right side, and injured the nose, though no fracture, not even of a cartilage, of the nose was found. The teeth were beautifully crowned with porcelain, backed by gold, so that the fractures were not perceptible to ordinary observation; he had a catarrhal conjunctivitis, granular and dry; asthenopia, with the slightest use of his eyes for the near; occasional insomnia; frontal headache; no appetite for breakfast, and was melancholy; he had never had rheumatism nor syphilis; did not drink, and retired early. He had been under treatment by several oculists, and had been observed by several dentists. Just before coming to me he had been under the care of a skilful oculist, who found occasion to interfere with the nares by repeated cauterization of the nasal mucous membrane. A weak lens had been worn, but the patient complained of its interference, and that it gave him no relief. Early in 1886, nausea and gastric disturbances were relieved by an astigmatic lens, but the astigmatism had disappeared, while the headaches had grown worse. On examination, I found the chief objective symptom to be the conjunctivitis; the nasal fossæ were amply large, and had no unusual discharge; the canine reflexes were intermittent, and shifting from one side to the other; and the teeth were, without exception, in perfect order. No glasses improved the vision, which for distance was perfect, and I looked upon the patient as suffering from invalidism, due to nervous reflexes, and treated him chiefly on general principles. Aside from a mild local astringent to the lids, his *régime* was a change in diet, more devotion to social pleasures, a trip to Washington, followed by ten days at the carnival in Montreal, etc. During these diversions he was much better as to the ocular symptoms, especially as to the bulbar pain, and his appetite returned. This improvement lasted for about two months, when the symptoms above mentioned recurred, and he sought other sources of relief. I have authentically learned since that a distinguished oculist had made a partial tenotomy of the internal rectus muscle of each eye, and that the patient was free from the incubus of nervous reflexes and had gained thirty pounds in weight.

This case is introduced to show the possibility of dental, nasal, and ocular reflexes concurring, and the elements in doubt in making an eliminative diagnosis. It further affords an illustration of the doctrine of the "survival of the fittest."

Before closing this paper, I desire to call attention to the dangers of overestimating reflex disturbances. That they exist, and that they are local expressions of a nerve disturbance, as suggested here of the fifth pair of nerves, cannot be denied. But that they are the necessary initial step of a future neurosis, such as chorea, epilepsy, or paralysis, is not true. It is always easy for the specialist to find symptoms that conform to a new and attractive theory of pathological phenomena; for new theories present an air of diversion and relief that is always refreshing. In the special subject under consideration, as in all references to nervous phenomena, it is a safe rule to keep before us the necessity of the healthy nutrition, both physical and moral, of the nervous system of our patients.

